# SynLethDB: synthetic lethality database toward discovery of selective and sensitive anticancer drug targets

**DOI:** 10.1093/nar/gkv1108

**Published:** 2015-10-29

**Authors:** Jing Guo, Hui Liu, Jie Zheng

**Affiliations:** 1 School of Computer Engineering, Nanyang Technological University, Singapore 639798, Singapore; 2 Lab of Information Management, Changzhou University, Jiangsu 213164, China; 3 Genome Institute of Singapore (GIS), Biopolis, Singapore 138672, Singapore

## Abstract

Synthetic lethality (SL) is a type of genetic interaction between two genes such that simultaneous perturbations of the two genes result in cell death or a dramatic decrease of cell viability, while a perturbation of either gene alone is not lethal. SL reflects the biologically endogenous difference between cancer cells and normal cells, and thus the inhibition of SL partners of genes with cancer-specific mutations could selectively kill cancer cells but spare normal cells. Therefore, SL is emerging as a promising anticancer strategy that could potentially overcome the drawbacks of traditional chemotherapies by reducing severe side effects. Researchers have developed experimental technologies and computational prediction methods to identify SL gene pairs on human and a few model species. However, there has not been a comprehensive database dedicated to collecting SL pairs and related knowledge. In this paper, we propose a comprehensive database, SynLethDB (http://histone.sce.ntu.edu.sg/SynLethDB/), which contains SL pairs collected from biochemical assays, other related databases, computational predictions and text mining results on human and four model species, i.e. mouse, fruit fly, worm and yeast. For each SL pair, a confidence score was calculated by integrating individual scores derived from different evidence sources. We also developed a statistical analysis module to estimate the druggability and sensitivity of cancer cells upon drug treatments targeting human SL partners, based on large-scale genomic data, gene expression profiles and drug sensitivity profiles on more than 1000 cancer cell lines. To help users access and mine the wealth of the data, we developed other practical functionalities, such as search and filtering, orthology search, gene set enrichment analysis. Furthermore, a user-friendly web interface has been implemented to facilitate data analysis and interpretation. With the integrated data sets and analytics functionalities, SynLethDB would be a useful resource for biomedical research community and pharmaceutical industry.

## BACKGROUND

Two genes are said to be in a synthetic lethality (SL) relationship if a perturbation of either gene alone is not lethal but perturbations of both genes lead to cell death or a dramatic decrease in cell viability (1). For example, the mutation of a given gene (a loss-of-function or gain-of-function defect) renders another gene essential so that this pair of genes form an SL relationship. Synthetic lethal interactions provide functional buffering and robustness, thereby enabling cells to maintain homeostasis in the face of diverse genetic and environmental challenges (2). By exposing the critical endogenous differences between cancer cells and normal cells, SL suggests a promising anticancer strategy. For instance, chemical inhibition of the SL partners of oncogenic genes would selectively kill cancer cells but spare normal cells (3). Therefore, SL-based therapeutics has the potential to overcome the drawbacks of traditional chemotherapies including severe side effects (4,5).

Since SL was first described in the studies on *Drosophila melanogaster* models (6), it has been most extensively explored in human and other model species. Two projects of genome-wide quantitative mapping of synthetic lethal interactions have been conducted for *Saccharomyces cerevisiae*, and the resulting SL networks provide valuable resources for understanding the functional relationships among genes (7,8). Recognizing the great potential of SL in anticancer therapies, researchers have developed experimental methods to detect SL interactions in cancer cells (9,10). For example, high-throughput pooled shRNA screening for gene essentiality has been developed, by which cell lines are infected with short hairpin RNA libraries targeting genome-wide mRNA. Then, the cells are cultured to allow the depletion of those cells containing shRNAs that target essential genes, after which synthetic lethal interactions can be identified by examining whether a gene is essential in the perturbed cell line but non-essential in the control cell line using microarray or deep sequencing (11).

However, the technology of pooled shRNA screening is still not able to cover the large number of genetic interactions that need to be surveyed across different cancer types so far. Hence, a few computational approaches have been proposed to complement the experimental screening for identifying SL interactions (12–14). Most *in silico* methods depend on comparative genomics to search for orthologous genes of the SL pairs in yeast that have been experimentally validated (14), or exploit other features such as evolutionary characteristics, metabolic networks and signaling pathways (15–17). Recently, a data-driven method, named DAISY, used the somatic copy number alterations, shRNA-based essentiality screens and co-expression patterns on hundreds of cancer cell lines to detect SL pairs in human (13).

With the increasing amount of SL-related data, a comprehensive database is urgently needed to gather SL gene pairs and relevant genomic and functional annotations. Also, the estimation of the druggability of SL gene pairs as drug targets and efficacy of inhibiting cancer cell viability is also important for the development of anticancer treatments. In this paper, we present SynLethDB, a comprehensive database dedicated to collecting SL pairs identified in various species, and integrating genomic and drug sensitivity data to conduct statistical estimation on druggability and efficacy. As a substantial extension of our previously proposed SL knowledge base, Syn-Lethality (18), we collected SL pairs from biochemical assays, other related databases, computational predictions and text mining results. For each SL pair, we computed a confidence score by integrating individual scores derived from different types of evidence. We also developed a statistical analysis module to estimate the druggability and efficacy of drug molecules for human SL pairs, based on genomic data (e.g. mutations, copy number alterations and gene expression profiles), drug–protein interactions and drug sensitivity profiles on more than 1000 cancer cell lines. To help users explore the wealth of data, we developed other practical functionalities, such as query and filtering, orthologous gene search, gene set enrichment analysis. Furthermore, we implemented a user-friendly web interface, including an interactive network and tabular viewer, statistical diagrams and graphical visualization plugins, to facilitate data display and interpretation. To the best of our knowledge, SynLethDB is the first comprehensive database that harbors a large set of SLs, and also contains data resources for systematic evaluation of SLs in anticancer drug discovery and development. We believe that SynLethDB would greatly facilitate and accelerate the discovery of selective and sensitive anticancer drug targets, based on the SL mechanism.

## SOURCES OF DATA

**Figure 1.**
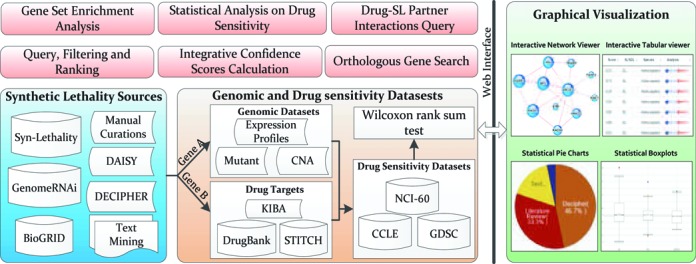
Schematic diagram of the data resources, functional modules and graphical visualization components included in SynLethDB. The SL sources include manual curations from publications, three related databases (Syn-lethality, BioGRID and GenomeRNAi), bi-specific SL shRNA screens (DECIPHER), computational predictions (DAISY) and text mining results. Genomic data (mutations, copy number alterations and gene expression profiles from COSMIC), drug targets (DrugBank, STITCH and KIBA) and three drug sensitivity data sets (CCLE, GDSC and NCI-60) are integrated, so that we can conduct Wilcoxon rank-sum tests to estimate the druggability and sensitivity of cancer cells upon drug treatments targeting human SL partners of genes mutated in the cancer cells. Six functional modules are developed to explore the data resources, and graphical visualization components are also implemented to facilitate data display and interpretation.

The first source of data in SynLethDB is the manually curated SL pairs from research papers concentrated on SL studies via biochemical experiments. Our previous SL knowledge base, Syn-Lethality (18), which contains manually collected SL pairs from the experimental literature, was integrated. Also, we collected SL pairs identified from high-throughput screening experiments, such as pooled shRNA screens, bi-specific shRNA screens (from the DECIPHER Project^1^), and combinatorial RNAi and drug screens. For the combinatorial RNAi and drug screening, the SL pairs were detected by conjugating the essential genes identified by RNAi with the drug's primary target genes deposited in DrugBank database (19). Secondly, a large number of genetic interactions annotated as SL pairs in BioGRID (20) were integrated into SynLethDB. Also, some gene pairs were annotated as SL in GenomeRNAi (21), a database devoted to collecting phenotypes from RNAi screens for *Drosophila* and *Homo sapiens*, and therefore these gene pairs have been added into our database. Thirdly, we included some human SL pairs computationally predicted by DAISY (13), in order to enrich our data set of human SL candidates that are potentially valuable for the discovery of anticancer drug targets. Figure 1 illustrates the various types of sources from which we collected SL pairs.

To extend the coverage of our database, we employed text mining tools to search for SL pairs that have been scattered in the literature. Using ‘synthetic lethal’ and ‘synthetic lethality’ as query keywords, we searched the whole PubMed database, and obtained 331 distinct publications with titles including either of the two keywords. As the contents of these publications focus on synthetic lethality, we used their abstracts as the training set to train the literature ranking tool MedlineRanker (22), which ranks the biomedical literature according to the relevance of a topic learned from the training set. The trained MedlineRanker was used to rank the PubMed publications published in recent 10 years, and the top 1000 publications were selected to conduct the following text mining procedures.

Next, we adopted PESCADOR (23), an information extraction tool for mining co-occurrences of concepts and gene/protein pairs from the literature, to extract gene/proteins associated with the concept of SL from the abstracts of the 1000 publications. In particular, the discriminative words identified by MedlineRanker, including ‘lethality’, ‘lethal’, ‘viability’, ‘apoptosis’, ‘cell death’, ‘synthetic lethality’ and ‘synthetic lethal’, were used as customized concepts that were taken as input by PESCADOR to discover concept-related word co-occurrences. According to the semantic structure of each sentence and the whole abstract, the genes/protein pairs co-occurring with the customized concepts are likely SLs reported in the literature. Furthermore, an appealing characteristic of PESCADOR is that the genes/protein pairs are categorized into four graded relevance degrees according to the scope (abstract or sentence) of the co-occurrence with the customized concept: genes/protein pairs and customized concepts co-occurring in an abstract (type 4), in a sentence (type 3), in a sentence with a biointeraction term (e.g. activates, induces, inhibits) (type 2) or in a sentence with a biointeraction term between the bioentity names (type 1). Based on the degree of relevance to the customized concepts, we regarded the genes/proteins pairs as SL and set their confidence scores to 0.2, 0,5, 0.7 and 0.9 for types 4, 3, 2 and 1, respectively. Finally, we manually curated the 337 PubMed publications whose titles include the terms ‘synthetic lethality’ or ‘synthetic lethal’, to ensure that we would not miss the SL pairs that have been explicitly reported by these studies.

In summary, the current version of SynLethDB contains 34 089 SL pairs that comprise 19 952 of *Homo sapiens*, 366 of *Mus musculus*, 423 of *Drosophila melanogaster*, 107 of *Caenorhabditis elegans* and 13 241 of *Saccharomyces cerevisiae*. More than 200 types of diseases and information of over 3314 publications have been deposited in SynLethDB. For each collected SL pair, we annotated its supporting evidence (e.g. mutations, RNAi screenings or predictions), species, diseases, references to PubMed and other relevant information, so that users can access the detailed information to explore the SL gene pairs. Furthermore, to prioritize SL pairs according to their reliability, we developed a scoring scheme to compute an integrative confidence score for each SL pair based on the annotations, as described in the following section.

## INTEGRATIVE CONFIDENCE SCORES

The SL pairs in our database were collected from different types of sources, including biochemical assays, other related databases, computational predictions and text mining results. Furthermore, biochemical assays were based on different experimental technologies and platforms, such as genetic mutation and transfection, RNA interference and drug inhibition. As multiple types of evidence contribute to the identification of a specific SL, an integrative confidence score combining scores from all these evidence sources can give an overall estimation of the reliability of an SL interaction. In principle, we assume that (i) experimental evidence contributes more significantly to the confidence score than evidence derived from predictive algorithms or text mining, and (ii) the SL pairs supported by more evidence sources should be given higher confidence scores than those supported by less evidence sources.

Due to the lack of a gold-standard set of SL pairs for validating the confidence scores, we aim to develop a scoring scheme that does not rely on comparison with any third-party data but rather relies on the available annotations associated with each SL pair. We developed a procedure of two steps, i.e. *quantification* and *integration*, to compute the confidence scores. A large number of SL pairs collected from wet-lab experiments and other related databases have only qualitative annotation evidence (such as ‘high-throughput’ or ‘low-throughput’), or technological descriptions about the wet-lab experiments (such as ‘shRNA screening’ or ‘mutation’), hence the quantification step is necessary to assign quantitative scores to those SL pairs before the calculation of integrative scores. Similar to the scoring scheme for protein–protein interactions (PPI) proposed by Cao *et al*. (25), we assigned the quantitative scores based on the experimental methods that were used to perturb SL partners, as shown in Table 1. For instance, ‘Mutant & Mutant’ means that the pair of SL genes are both perturbed via mutations induced by transgenic or genetic deletions, and ‘RNA interference & Mutant’ means that one gene is perturbed by RNAi and the other is perturbed via mutation. In general, results from low-throughput experiments, due to a lower false positive rate, are considered to be more reliable than results from high-throughput experiments, hence we assigned a higher confidence score to low-throughput evidence than high-throughput evidence. RNA interference experiments, such as shRNA, siRNA and dsRNA, frequently manifest considerable variability in knockdown efficacy and off-target effects; drug inhibitors also tend to show limited inhibition on target proteins and off-target effects which may lead to false positives. Accordingly, they are assigned relatively low confidence scores compared to the scores of mutation or transfection experiments.

**Table 1.** Quantitative scores assigned to SLs according to the experimental methods annotated in evidence sources

Experimental method	Score
Mutant & Mutant	0.90
RNA interference & Mutant	0.75
Bi-specific RNA interference	0.50
RNA interference & Drug inhibition	0.50
Low-throughput	0.80
High-throughput	0.50

If there exist multiple pieces of evidence of the same type (e.g. experimental evidence) supporting a specific SL pair, we adopted the probability disjunction formula to combine the individual scores as follows:

(1) \begin{equation*} s = 1-\prod _{i=1}^n(1-p_i), \end{equation*}

in which *s* represents the integrative score corresponding to the experimental evidence, *p*_*i*_ is the individual score and *n* is the total number of pieces of experimentally supporting evidence. For example, an SL with one ‘RNA interference & Mutant’ evidence and one ‘bi-specific RNA interference’ screening evidence will lead to the combined score of 0.875, i.e. 1 − (1 − 0.75)(1 − 0.5) = 0.875. Note that the probability disjunction formula has been frequently used to calculate combined scores in the case that multiple pieces of evidence exist, such as in STITCH (26) and ComPPI (27).

In the *integration* step, we introduced weight factors to reflect the importance of different types of evidence. To obtain a normalized score between 0 and 1, such that a score closer to 1 represents higher confidence, we computed the normalized weighted sum as:

(2) \begin{equation*} S = \frac{w_ms_m+w_ds_d+w_ps_p+w_ts_t}{w_m+w_d+w_p+w_t}, \end{equation*}

in which *S* represents the integrative confidence score; $w$_*m*_, $w$_*d*_, $w$_*p*_ and $w$_*t*_ are the weight factors of biochemical experiment, other related databases, computational prediction and text mining-based evidence; *s*_*m*_, *s*_*d*_, *s*_*p*_ and *s*_*t*_ are corresponding individual scores. Following the convention that evidence from biochemical experiments is the most reliable, followed by other related databases and *in silico* predictions, and text mining-based evidence is the least reliable, we set the weight factors $w$_*m*_, $w$_*d*_, $w$_*p*_ and $w$_*t*_ to 0.8, 0.5, 0.3 and 0.2, respectively.

## STATISTICAL ANALYSIS OF DRUG SENSITIVITY

Although a perturbation of an SL pair via genetic mutation or RNAi inhibition can induce cell death with a high probability, it is likely that only low sensitivity or even no lethal response upon drug treatments can be observed. A reason may be that the proteins encoded by the SL parters are not accessible to drug molecules (i.e. lack of druggability), or their biological functions are not completely blocked by small drug molecules (i.e. low efficacy). Insufficient response to drug treatments could hinder the practical application of the SL concept to anticancer drug design.

To give a preliminary evaluation of the SL pairs as potential anticancer drug targets, we developed a statistical analysis module to evaluate the druggability and efficacy of SL pairs upon drug treatments, based on the large-scale drug sensitivity data sets. In particular, we built a set of high-quality drug–protein interactions from the drug targets in DrugBank (19), drug–protein interactions with experimentally supportive scores >0.9 in STITCH (26), and the drug–kinase binding affinity profiles, referred to as KIBA (28), which were integrated from three drug bioactivity assays (29–31) and ChEMBL (32). We also integrated three large-scale drug sensitivity data sets, i.e. CCLE (33), GDSC (34) and NCI-60 (35), together with genome-wide gene expression profiles, copy number alterations (CNA) and mutations obtained from the Catalogue of Somatic Mutations in Cancer (COSMIC) database (36). Overall, these data sets contain drug sensitivity values (represented as the half maximal inhibitory concentration values, i.e. IC50) of 19 017 unique approved and experimental drugs on more than 1000 cancer cell lines. The large amount of data allows us to carry out powerful statistical tests to examine whether a specific SL can induce significant cancer cell death or reduce cancer cell viability when perturbed by a drug. Formally, for each SL pair, denoted as *A* and *B*, a Wilcoxon rank sum test can be conducted to examine if inhibiting gene *B* by drugs yields significant drug sensitivity levels in samples in which gene *A* is inactive (or overactive) than in the rest of the samples. It is worth noting that such a statistical test was also used by the DAISY method to detect SL pairs from somatic copy number alterations and shRNA essentiality screening data (13).

## FUNCTIONALITIES

**Figure 2.**
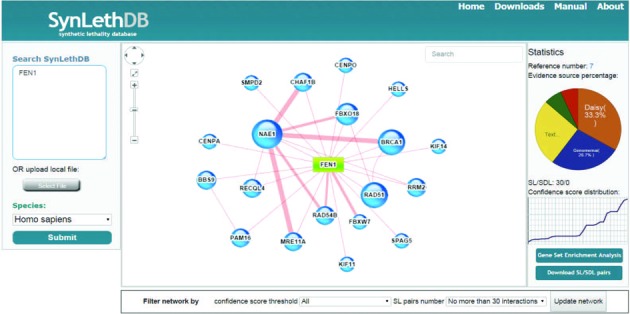
Screenshot of the main page of the SynLethDB database which displays the search result of the query gene Fen1 on human. This network shows all human SL pairs collected by our database. Users can update the network by set a different threshold for the confidence score and the number of SL pairs to be displayed via the network viewer. On the right part of the page, statistics about the percentages of evidence sources, reference number and confidence score curve are displayed.

**Figure 3.**
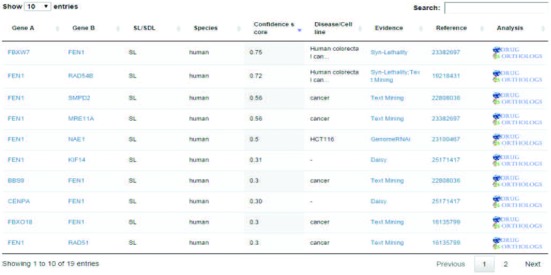
Screenshot of a tabular viewer that displays all the SL partners of Fen1 deposited in SynLethDB, along with the corresponding evidence sources, species, diseases, confidence scores and PubMed references associated with each SL pair. Users can rank the SL pairs according to integrative confidence scores by clicking the column name. Also, one click can launch the statistical analysis of the responses of cancer cells upon drug treatments targeting human SL genes.

We have developed six functional modules to help users explore the wealth of data. The *query*, *filtering* and *ranking* module take as input one or more gene names to search for all associated SL partners, and the SL pairs are represented in the form of both network and tabular viewers. To provide users with a biological context, the network also includes the SL relationships between the genes associated with query genes. In the network viewer, the widths of the edges are proportional to the integrative confidence scores corresponding to the SL pairs, and users can filter the query results by specifying different thresholds of the confidence score and numbers of SLs, as shown in Figure 2. Each gene is linked to public resources such as UniProt (37), Ensembl (38) and NCBI GenBank (39). In the tabular viewer, the species, diseases and integrative confidence scores are displayed for each SL pair. Detailed information about the evidence sources and individual scores can be displayed by clicking the hyperlinks of evidence sources. With the *ranking* function of the tabular viewer, users can easily pick up high-confidence SL pairs according to the integrative confidence scores, as shown in Figure 3.

As comparative genomic analysis has been successfully used to predict SL by searching for orthologous genes across species, we collected the orthologs among the five organisms identified by four leading methods, i.e. InParanoid (release 8.0) (40), HomoloGene^2^ (build68), Ensembl Compara (41) and PhylomeDB v4 (42). The four methods differ from each other in the underlying rationales for orthology inference and thus complement each other, allowing us to construct a comprehensive set of orthologs (43,44). For any SL pair of interest in one species, users can search for the orthologous genes in the other four species. This functionality could potentially extend the coverage of our SL database. Particularly, if any pair of orthologs found in other species has been already annotated as SL, this could strengthen our confidence in the SL pair, although currently we have not yet considered its contribution to the integrative confidence score.

For human SL pairs, we developed the *statistical analysis of drug sensitivity* functional module to test the druggability and efficacy to drugs targeting SL partners based on the collected large-scale drug sensitivity data sets. For each SL pair, one click can launch the statistical analysis procedure and the statistical significance (measured by *P*-value) will be calculated. To facilitate data interpretation, graphical representations with interactive features, such as scatter plots, statistical boxplots and scatter plots, are employed. In these graphical plots, drug names, sensitivity values and cancer cell lines are interactively displayed. Also, the drugs targeting the SL partners of interest can be viewed via the *drug-SL partner interaction query* functionality. All displayed drugs are linked to the PubChem database (45) which provides detailed properties and chemical structures.

Furthermore, as *gene set enrichment analysis* (GSEA) is helpful for understanding the molecular mechanisms of SL interactions, we carried out gene set enrichment analysis to find statistically significant pathways and GO (gene ontology) functional annotation terms, based on the subset of genes constituting SL relationships with each specific gene. For the identified pathways and GO terms, links to external databases, such as KEGG (46), Reactome (47) and Gene Ontology (48), are provided.

## CONCLUSION AND FUTURE DEVELOPMENT

In this paper, we proposed SynLethDB, a comprehensive database of synthetic lethality. SL pairs were collected from multiple sources, including biochemical assays, other related databases, computational predictions and text-mining outputs for five species. To extend the coverage of SL gene pairs, we adopted text mining tools to analyze the PubMed literature related to synthetic lethality. To facilitate the data interpretation and evaluation, we developed useful functional modules such as orthology search, query and filtering, statistical analysis on drug sensitivity and gene set enrichment analysis, etc. As the first comprehensive database dedicated to synthetic lethality, which is an emerging anticancer strategy promising to be selective and sensitive, SynLethDB can be a valuable resource to facilitate the discovery of new anticancer drug targets.

In future, we will expand the coverage of data types and species, on the basis of a rapidly increasing numbers of studies focused on SL screening and sensitivity analysis of cancer cells to drugs. We will continuously increase the number of manually curated SL pairs to ensure the reliability of data, and build a gold standard for human SL, which would be very helpful for biomedical research community in validating and evaluating results produced with both experimental and computational approaches. In addition, we will incorporate new SL pairs from other sources, such as more computational predictions and text mining results, to complement the manual curations.

Furthermore, it has been realized that the cellular response of cancer cells to drug treatments depends strongly on the genetic context, such as spectrum of mutations, copy number alterations and epigenetic modifications (49). We will go on to identify cancer-specific SL pairs by integrating the genomic and epigenetic features into our database. Also, we will develop more functional modules and data visualization tools to analyze and display the data.

## Footnotes

1 http://www.decipherproject.net/shRNA-libraries/bi-specific/

2 http://www.ncbi.nlm.nih.gov/homologene/
